# Evaluating the Topological Features of Monomeric and Trimeric TRAF2-C: A Multi-Disciplinary Approach

**DOI:** 10.3390/biom15111626

**Published:** 2025-11-19

**Authors:** Fulvio Erba, Daniela Russo, Velia Minicozzi, Luisa Di Paola, Sylvain Prevost, Anastasia De Luca, Giampiero Mei, Almerinda Di Venere

**Affiliations:** 1 Department of Clinical Science and Translational Medicine, Tor Vergata University of Rome, Via Montpellier 1, 00133 Rome, Italy; erba@uniroma2.it; 2 CNR-Istituto Officina dei Materiali, c/o Institut Laue Langevin, 71 Avenue des Martyrs, CS 20156, CEDEX 9, 38042 Grenoble, France; 3 Department of Physics, Section of Roma Tor Vergata, National Institute for Nuclear Physics (INF), University of Rome Tor Vergata, Via della Ricerca Scientifica 1, 00133 Rome, Italy; 4 Unit of Chemical-Physics Fundamentals in Chemical Engineering, Department of Engineering, University Campus Bio-Medico of Rome, Via Álvaro del Portillo 21, 00128 Rome, Italy; 5 Large Scale Structures, Institut Laue-Langevin，71 Avenue des Martyrs, CS 20156, CEDEX 9, 38042 Grenoble, France; prevost@ill.fr; 6 Department of Biology, University of Rome Tor Vergata, Via della Ricerca Scientifica 1, 00133 Rome, Italy; 7 Department of Experimental Medicine, University of Rome Tor Vergata, Via Montpellier 1, 00133 Rome, Italy

**Keywords:** TRAF2-C, oligomerization, protein dynamics, TNF receptor signaling

## Abstract

This study investigates the structural dynamics of the TRAF2 C-terminal domain (TRAF2-C), a key adaptor protein in TNF receptor signaling. TRAF2 usually forms trimers, but its ability to dissociate into monomers is critical for regulating apoptosis, inflammation, and cell survival. Using Fluorescence Fluctuation Spectroscopy, dynamic light scattering, circular dichroism, and Small Angle Neutron Scattering, we analyzed TRAF2-C over a wide concentration range. At nanomolar levels, the protein dissociates easily, with trimers representing only a minor fraction, while micromolar concentrations strongly favor trimerization. Dissociation also reduces α-helical content without disrupting the overall fold. Molecular dynamics simulations and protein contact network analysis support this analysis, identifying interfacial residues and hydrogen bonds as key factors stabilizing oligomers and enabling dynamic asymmetry. Overall, these findings highlight TRAF2-C’s capacity to switch between monomeric and trimeric states as a crucial regulatory mechanism, offering insights into TRAF-mediated signaling and potential therapeutic strategies.

## 1. Introduction

**Figure 1:**
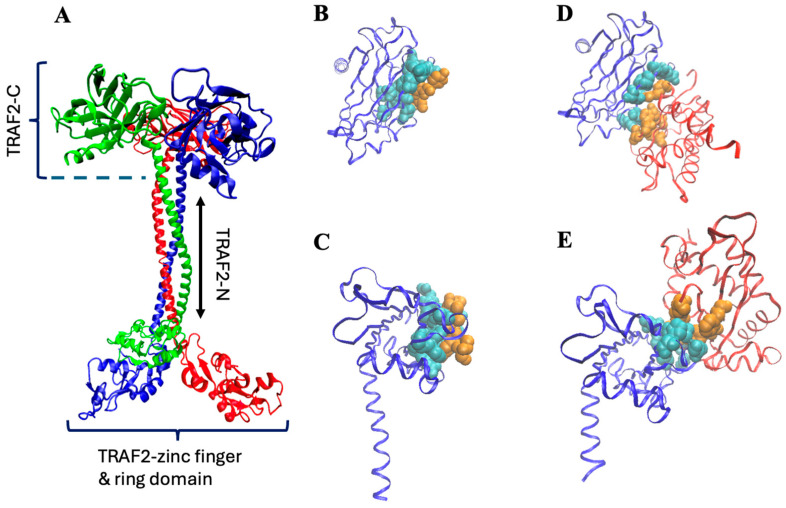
(**A**) Trimeric TRAF2 shape obtained through the available crystallographic models of the C-terminal, coiled coil, and zinc-finger/ring sections (pdb: 5et1, 3m0a, 3knv, respectively). (**B**,**C**) monomeric TRAF2-C (blue ribbon) interacting with the CD40 peptide ((**B**), top view; (**C**), side view; pdb file 1qsc). (**D**,**E**) Monomeric TRAF2-C interacting with TRADD N-terminal ((**D**), top view; (**E**), side view; pdb file 1fv3). The protein residues involved in the interaction are in cyan, while those of the ligand are in orange. In (**D**,**E**), the remaining structure of TRADD is reported in red.

TNF receptor-associated factors (TRAFs) are members of a group of proteins that play multiple roles in cellular response, mediating the signal transduction from receptors to the downstream signaling cascades [1]. Thanks to a peculiar common structure [2], TRAFs act as adaptor molecules, binding a number of different proteins, including receptors (TNFRs) and the inhibitors of apoptosis (cIAPs) [2,3,4]. TRAF2, in particular, is the main mediator of TNF-a signaling, controlling both the canonical and non-canonical NF-κB pathways [5,6], thus indirectly regulating the production of inflammatory molecules [7]. In addition, TRAF2 shares with other members of the TRAFs family an N-terminal ring section that confers the protein further important functional roles. For instance, thanks to the ring domain, TRAF2 displays also an E3-ubiquitin ligase activity [8,9] that allows polyubiquitination of caspase-8 and, as a consequence, the prevention of apoptosis [10]. Being involved in such a variety of biological processes, TRAFs are principal regulators of cell death and survival, and their malfunctioning can be the source of severe pathologies. Inflammation, cell malignancies, and carcinomas are examples of TRAFs-related diseases [11], thus explaining why such proteins have become crucial targets for therapeutic intervention [12]. Indeed, in the last five years, hundreds of papers published in the literature deal directly or indirectly with TRAFs activities. Despite such efforts, the molecular mechanism of these proteins is poorly understood, because a complete crystallographic model is not yet available. Most of the members of the TRAF family are assembled as homo-trimers whose monomeric subunits share a common domain organization (Figure 1A): a globular carboxy-terminal domain (TRAF-C, residues 355–501) and a long coiled-coil tail (TRAF-N, residues 271–335), which ends with a third, more flexible section, composed by zinc-finger motifs (residues 98–271) and a ring (residues 1–98) domain [2]. The TRAF-C and the TRAF-N sections form the so-called TRAF domain, whose crystallographic structure has been only partially solved (residues 334–501) and purified (residues ≈ 310–501). The role of the globular C-terminal domain is particularly important in the case of TRAF2. For instance, it can bind glutathione transferase P1-1 [13], forming a complex that sequestering TRAF2 may inhibit apoptosis in tumor cells [14]. A more important function of TRAF-C is its capacity to interact with TNF receptors. Depending on the specific kind of TNF involved, TRAF2-C can, in fact, directly bind to TNFR2, CD40, and CD30, while it requires the TRADD adaptor protein to interact with TNFR1 (Park, 2018 [2]). Structural studies have demonstrated that the direct interaction of TRAF2-C with CD40 receptor peptides [15] and/or with the TRADD adaptor protein [16,17] covers a similar area on the protein surface (Figure 1), but the number (and typology) of contacts is different in the two cases [17]. Modeling of TRAF2-C dynamics [18] has revealed that the asymmetric movements of the three subunits affect the protein surface, thus probably having a major role in the modulation of TRAF2-C interactions with both the membrane receptor(s), and with the TRADD adaptor protein. In this context, the length of the TRAF2 plays an important functional activity too. In fact, the dynamic asymmetry at the subunit interface of TRAF2-C is considerably reduced if a longer TRAF-N section is considered [19], suggesting that the coiled-coil tail might influence the protein quaternary interactions at the level of its globular domain (Figure 1A).

The equilibrium between monomeric and oligomeric TRAFs states is important for several other reasons. For instance, TRAF2 can generate a heterotrimeric complex with TRAF1 [4] to bind the cellular inhibitors of apoptosis (CIAPs), which participate in NF-κB signaling, regulating apoptosis and inflammation [20,21]. Furthermore, TRAF2 and other members of the TRAF family may form homo- and hetero-dimers of trimers through their ring domains, a process that is crucial in the regulation of TRAF-mediated signaling [9,22]. Finally, in the specific case of TRAF2-C, it was discovered that the equilibrium between monomeric and trimeric states strongly influences the binding to synthetic [23] and natural membranes [24], causing vesiculation and/or membrane fusion [24,25]. For all these reasons, the study of the structural properties of the TRAF2 quaternary interactions is crucial to understanding whether it is possible to modulate TRAF2 activities by interfering with the protein monomer–trimer equilibrium. In previous studies on the TRAF2-C, it has been demonstrated that this trimeric portion of TRAF2 can be easily dissociated into monomeric subunits, in vitro, using high hydrostatic pressure [26], decreasing the protein concentration [26], or lowering the pH of the buffer [25]. Molecular dynamic (MD) simulations on monomeric TRAF2-C suggested that monomerization only partially affects the protein secondary structure, with most changes occurring in the a-helix content [26]. Such prediction has been recently confirmed by dissociating the protein at pH 5.5 and measuring the secondary structure content using circular dichroism spectroscopy [25].

Taking advantage of the extremely high sensitivity of Fluorescence Fluctuation Spectroscopy (FFS) at very low concentration, in the present study, we investigated in detail the dissociation of TRAF2-C in the nanomolar range (2–50 nM). Circular dichroism (CD), dynamic light scattering (DLS) and Small Angle Neutron Scattering (SANS) have been instead employed to characterize the protein structural features at higher concentration (1–20 mM). The combination of these techniques allowed us to span a range of four orders of magnitudes (2 nM–20 mM) and to obtain an estimation of the equilibrium constant, assuming a T3 ↔ 3M equilibrium. In parallel, MD simulations have been performed on different TRAF2-C association states to assess the impact of quaternary structure interactions on the overall protein shape. The equation of an ellipsoid of revolution has been used to fit the simulated data and to obtain the corresponding principal axes values, which resulted in good agreement with the experimental results of SANS experiments. Finally, a protein contact network (PCN) was used to analyze the frames obtained in MD and to identify which residues are more involved in the dynamic at the subunits’ interface. The combination of these experimental and theoretical results provides for the first time a detailed description of the tridimensional shape of TRAF2-C in solution and of its multiple (monomer–dimer–trimer) aggregation states.

## 2. Materials and Methods

### 2.1. Protein Expression and Purification

The C-terminal domain of human TRAF2 (TRAF2-C, residues 310–501) was cloned into a pET-based expression vector and overexpressed in *Escherichia coli* BL21 (DE3) cells. Protein expression was induced with 0.5 mM IPTG at 18 °C for 16 h. Cells were harvested and lysed by sonication in a buffer containing 50 mM Tris-HCl (pH 7.5), 150 mM NaCl, and 1 mM DTT. The lysate was clarified by centrifugation at 15,000× *g* for 30 min and the supernatant was loaded onto a Ni-NTA affinity column. After washing, TRAF2-C was eluted using a linear imidazole gradient. Protein purity (>95%) was assessed by SDS-PAGE, and buffer was exchanged using size-exclusion chromatography on a Superdex 75 column equilibrated with 20 mM Tris-HCl, 100 mM NaCl, pH 7.4. Final monomer protein concentration was determined spectrophotometrically using ε_280_ = 29,910 M^−1^ cm^−1^. Alexa Fluor 488 and rhodamine 6G were purchased from SIGMA ALDRICH.

### 2.2. Spectroscopic Technique

#### 2.2.1. Fluorescence Correlation Spectroscopy (FCS)

FFS, FCS, and photon counting histogram (PCH) measurements were performed using the ISS-ALBA fluorescence correlation spectrometer (ISS, Champaign, IL, USA) equipped with a Nikon inverted microscope. Two-photon excitation (in the range 780–800 nm) was provided by a Ti/sapphire mode-locked laser (Chameleon Ultra; Coherent Inc., Santa Clara, CA, USA). The instrument alignment was performed using a dilute solution (~10 nm) of 6G rhodamine. Protein samples were labeled with Alexa Fluor 488 at a 1:1 stoichiometry. FCS measurements were conducted at 20 °C in phosphate buffer (PBS, pH 7.4) at protein concentrations ranging from 4 to 60 nM. Autocorrelation functions and PCH data were fitted using ISS-Vista software version 3.0. The points of autocorrelation were analyzed with a Gaussian–Lorentzian intensity profile distribution, while for PCH the estimation of brightness was indicative of the relative abundance of monomers and trimers.

#### 2.2.2. Dynamic Light Scattering (DLS)

DLS measurements were carried out on a Horiba (Kyoto, Japan) LB-550 nanoparticle size analyzer at 20 °C. Samples (in terms of monomers) at concentrations of 0.1–4 µM were filtered (0.22 µm) and measured in triplicate. Hydrodynamic diameter distributions were obtained by the accompanying software based on a Fourier-transform deconvolution procedure and then fitted with a bimodal Gaussian distribution to assess the relative proportions of monomeric and trimeric species. The particle dispersity was then calculated using the following expression:

$$\mathrm{particle}\;\mathrm{dispersity}\;=\;\frac{\sum_{i}{\left(D-<D>\right)}^{2}q(i)/100}{{<D>}^{2}}$$

where $<D>=\sum_{i}D_{i}q(i)/100$, the $q\left(i\right)$ being the experimental DLS distribution.

#### 2.2.3. Circular Dichroism Spectroscopy

Far-UV CD spectra were recorded on a Jasco J-815 spectropolarimeter at 20 °C, using a 1 mm pathlength quartz cuvette. Spectra were acquired from 200 to 250 nm, at protein concentrations ranging from 0.25 to 33 µM. The data were corrected for buffer contributions and converted to molar ellipticity. Secondary structure contents were estimated using the BeStSel server [27], and the relative α-helix and β-sheet content was quantified at each dilution point.

#### 2.2.4. Small Angle Neutron Scattering (SANS)

Small Angle Neutron Scattering (SANS) data were measured on D11 at the Institut Laue-Langevin—The European Neutron Source (ILL, Grenoble, France) operating at 44 MW with a multi-PSD ^3^He detector, at a constant wavelength λ of 4.6 Å (relative fwhm 9%), at 2 sample-to-detector distances of 1.7, 16.5, covering a q-range of 0.004–0.6 Å^−1^, where *q* = (4π/λ)sin(θ) is the magnitude of the wavevector, 2θ being the scattering angle. Samples were kept in quartz cuvettes of 1 mm pathway (type 100-QS, Hellma GmbH, Müllheim, Germany) on a thermalized sample-changer at 25 °C. Data were reduced using Grasp V10.34c [28], normalizing the intensity using the measured direct beam flux with a calibrated attenuator. Mid and high Q data were merged and analyzed using Sasfit program in the Q range 0.01–0.22 Å^−1^.

### 2.3. Molecular Dynamics Simulations

We performed classical molecular dynamics simulations of the TRAF2-C monomer, dimer, and trimer, starting from the X-ray diffraction structure (Protein Data Bank entry 1CA4), using the GROMACS simulation package [29,30,31,32] and the GROMOS43A1 force field [33], to ensure comparability with previous simulations [26,34]. All simulations were carried out in the NpT ensemble.

The temperature was maintained at 293 K using the v-rescale thermostat [35] with a coupling constant of 0.1 ps. The pressure was kept at 1 bar using the Berendsen barostat [36], with a coupling constant of 1 ps and an isothermal compressibility of 4.5 × 10^−5^ bar^−1^. Water molecules were described using the simple point charge (SPC) model.

The simulation box was cubic, with side lengths of 7.0 nm, 9.7 nm, and 17.6 nm for the monomer, dimer, and trimer, respectively. The systems were solvated with 10,524, 28,223, and 176,492 water molecules, respectively. An appropriate number of Na^+^ or Cl^−^ counterions was added to ensure overall charge neutrality. Simulations were carried out at neutral pH.

Periodic boundary conditions were applied in all directions. Long-range electrostatic interactions were treated using the particle mesh Ewald (PME) method [37]. A 2 fs time step was used, with a non-bonded pair list cut-off of 1.4 nm, updated every 10 steps.

Each system was first energy-minimized in vacuum using the steepest descent algorithm. Counterions and water molecules were then added. The solvent was equilibrated separately with a short 10 ps NVT run at 200 K, while the solute was kept fixed. Subsequently, the entire system was equilibrated in the NVT ensemble for 50 ps at 293 K. Production MD simulations were then run in the NpT ensemble for 200 ns (monomer), 240 ns (dimer), and 500 ns (trimer), all at 293 K.

### 2.4. Analysis of Simulated Data

#### 2.4.1. Evaluation of the Ellipsoid of Revolution of Trimeric and Monomeric TRAF2-C

According with http://www.juddzone.com/ALGORITHMS/least_squares_3D_ellipsoid.html (accessed on 9 June 2025), atomic coordinates of monomeric and trimeric TRAF2-C have been used as input on a modified Python v3.11.4 script. The code includes a least square fit of the following general ellipsoid equation:

Ax^2^ + By^2^ + Cz^2^ + Dxy + Exz + Fyz + Gx + Hy + Iz = 1

being x, y, and z the coordinates of the different structures. The fitted coefficients A, B, C, D, E, F, G, H, and I, thus have been used to calculate the center of ellipsoid, the length of axes, and the rotation matrix with respect to origin. Both the monomeric than the trimeric structure of TRAF2-C have been displayed using Pymol v 3.0 (The PyMOL Molecular Graphics System, Schrödinger, LLC, New York, NY, USA) superimposed with the respective fitted ellipsoid.

#### 2.4.2. Fractal Dimension

In order to assess how the protein superficial roughness changes in time along the MD simulation, fractal dimension (FD) of trimeric, dimeric, and monomeric structures have been considered.

The following equation (Lewis et al., 1985 [38]) has been used to calculate FD:

$$2-FD=\frac{dlog\left(SASA\right)}{dlog\left(PR\right)}$$

being SASA the Solvent Accessible Surface Area and PR the probe’s radius, respectively. An array of PR ranging from 1.0 Å to 2.0 Å (step 0.2 Å) has been used in a Python script to calculate SASAs with different sensitivity. Thus, FD has been calculated fitting log (SASA) vs. log (PR) using a linear regression algorithm for the t = 0, t = 150, and t = 200 ns MD’s frames, respectively.

#### 2.4.3. Protein Contact Networks and Network Clustering

Protein contact networks are a coarse-grained representation of protein structures as networks [39], where single residues are the network nodes, while links are the noncovalent intramolecular interactions between residues, sorted out by mutual distance (Euclidean distance between residues’ α-carbons).

The Adjacency Matrix mathematically defines the network, such as

$$A=\{\;\begin{array}{c} 1\;if\;{4\;Å<d}_{ij}<8\;Å\\ 0\;otherwise \end{array}$$

A node’s degree represents how many links connect it to other nodes.

Protein contact networks can be parted into clusters, groups of nodes identified by maximizing intra-cluster links when compared to inter-cluster. This method applies to the PCNs Laplacian matrix *L* defined as

L=D−A

*A* is the adjacency matrix, *D* is the degree matrix (a diagonal matrix whose diagonal is the degree vector). Applying the spectral decomposition to the Laplacian *L*, the eigenvector $v_{2}$ corresponding to the second minor eigenvalue, known as the Fiedler vector, is used for the clustering partition [39]. The different clusters are represented as Clustering Color Maps, allowing a fast identification of different clusters along the sequence.

## 3. Results and Discussion

### 3.1. In Silico Prediction of TRAF2-C Superficial Roughness

**Figure 2:**
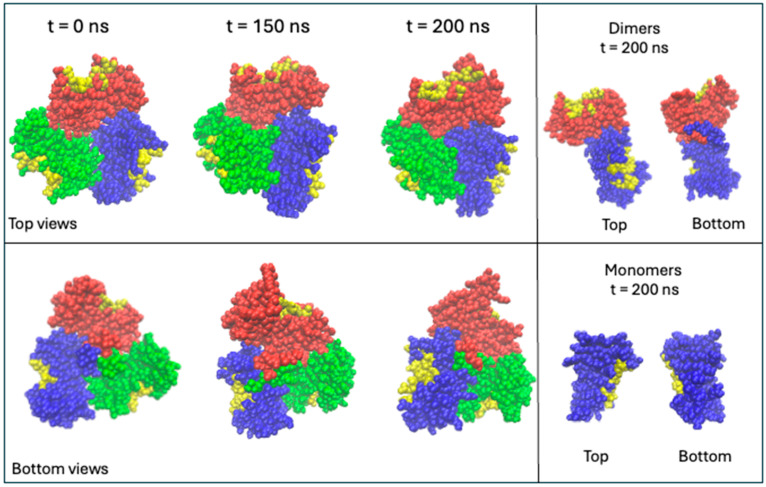
Top and bottom views of crystallographic TRAF2-C and simulated TRAF2-C at 150 and 200 ns. On the right side of the figure, the simulated dimeric and monomeric proteins at 200 ns are reported. The three chains are colored in blue (subunit A), red (subunit B), and green (subunit C). The TRADD and CD40 binding residues are reported in yellow.

In a previous study, molecule docking was used to mimic TRAF2-C binding to peptide receptor [18]. The simulation suggested that the reciprocal interaction of the three subunits might strongly influence the ligand binding process. In particular, the displacement of the monomers produced an asymmetric configuration both in the quaternary assembly and at the level of the protein surface [18]. At this regard, simulations of different TRAF2-C forms have been carried out to predict the extension of structural superficial changes due to the dynamics occurring in an aqueous environment. The structure of trimeric TRAF2-C obtained after 150 or 200 ns is reported in Figure 2, along with the X-ray crystallographic model (pdb:1ca4), which was used as a frame of reference. The effect on the surface of the reciprocal movements of the three subunits on the TRADD and CD40 binding site has been highlighted at 0, 150, and 200 ns, indicating in yellow the residues involved in such interaction, as already mentioned in the introduction, and shown in Figure 1 (for an analysis of RMSD see Section 3.5). The evolution of smaller oligomeric forms (dimers and monomers) has also been characterized by MD simulations and the results at 200 ns are reported in Figure 2 (right panels).

**Figure 3:**
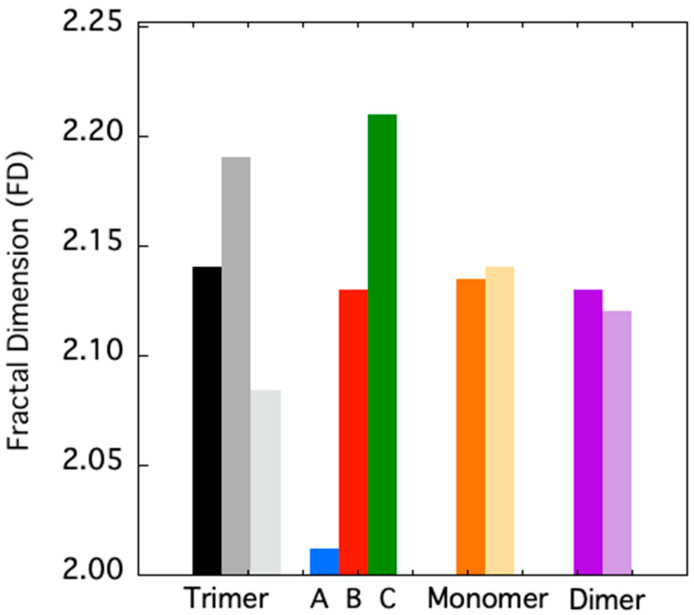
FD of trimeric TRAF2-C at 0 ns (black-filled bar), at 150 and 500 ns (dark and light gray bars). The FD of the three subunits analyzed separately at 500 ns are reported in blue (subunit A), red (subunit B), and green (subunit C). The FD values of the simulated TRAF2-C monomer (orange) and dimer (purple) structures at 0 and 200 ns are shown in dark and light colors, respectively.

To quantitatively assess the extension of such “dynamic asymmetry”, we studied the evolution of the TRAF2-C fractal dimension at different simulation times. This approach has become very useful to characterize some topological features of biological macromolecules. In particular, the FD of globular proteins resulted to be in the range 1.7–2.3 [38,40]. As shown in Figure 3, initially the FD values of trimeric, dimeric, and monomeric TRAF2-C are quite similar, being 2.14 and 2.12 and 2.13, respectively. In the case of the trimer, the overall FD increases up to ≈2.19 at t = 150 ns and then decreases to ≈2.08, at t = 500 ns. Interestingly, if the three subunits are taken apart and analyzed separately, at 500 ns a strong asymmetry in the surface roughness of the monomers is observed (Figure 3), the FD value spanning from 2.01 (subunit A) to 2.21 (subunit C). The MD of monomeric and dimeric TRAF2-C at 200 ns display smaller changes with respect to that observed at t = 0 ns, thus confirming that the larger fluctuation observed in the trimer is peculiar of that oligomeric configuration and arises from the reciprocal interaction of the three subunits.

### 3.2. FFS Determination of TRAF2-C Diffusion Coefficients and Particle Size Distribution

**Figure 4:**
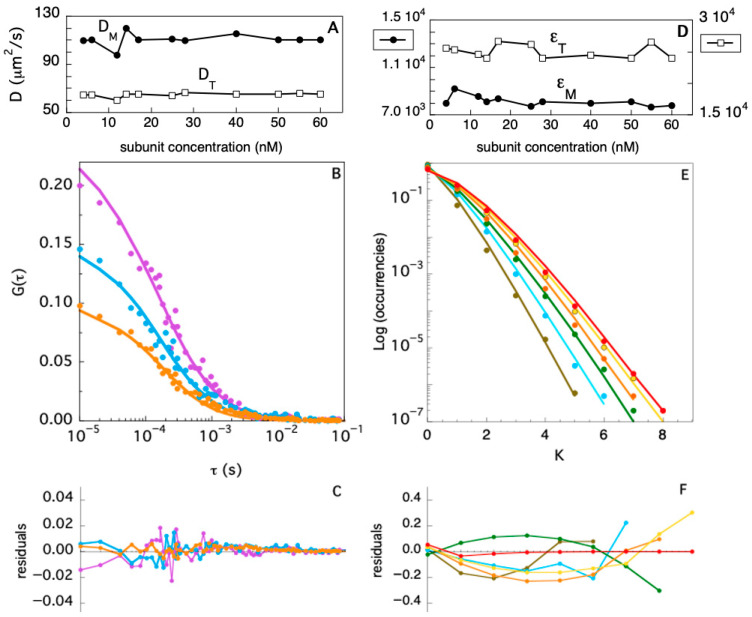
(**A**) Monomer (D_M_) and trimer (D_T_) diffusion coefficients evaluated fitting FCS autocorrelation functions at different TRAF2-C concentrations. Examples of autocorrelation functions, fits, and residuals are reported in (**B**,**C**) for [TRAF2-C] ≈ 12, 17, and 40 nM (purple, cyan, and orange symbols and lines, respectively). (**D**) Monomer (ε_M_) and trimer (ε_T_) brightness evaluated fitting PCHs at different TRAF2-C concentrations. Examples of PCHs, fits, and residuals are reported in (**E**,**F**) for [TRAF2-C] ≈ 4, 12, 25, 40, 50, and 55 nM (brown, cyan, green, orange, yellow, and red symbols and lines, respectively).

One important question about globular, oligomeric proteins is whether they retain a native tertiary structure upon dissociation into monomeric species. This information is particularly important in the case of multiple ligands- and receptors-binding proteins such as the members of the TRAF family. Insights on the size and the shape of a large biological molecule can be obtained determining the value of its diffusion coefficient, D [41]. The experimental determination of TRAF2-C diffusion coefficient has been carried out using Fluorescence Fluctuation Spectroscopy (FFS), which has become a powerful and robust technique to study the equilibrium dissociation of oligomeric proteins [42,43]. In particular, the development of confocal microscopy and Fluorescence Correlation Spectroscopy (FCS) have opened in recent years new perspectives on the characterization of protein conformational dynamics and diffusion in solution, on membranes, and in vivo [44]. In order to characterize the TRAF2-C dissociation process, we have performed several FCS measurements in buffer, at 20 °C, varying the protein concentration. Three typical autocorrelation functions, extrapolated from FFS measurements, are reported in Figure 4B, at three different subunit concentrations ([M] ≈ 12 nM, [M] ≈ 17 nM, and [M] ≈ 40 nM). The data have been fitted fixing the total monomer concentration (obtained from spectrophotometric assays) and using two variable diffusion coefficients, D_M_ and D_T_, for the monomer and the trimeric species, respectively. The initial values of the two parameters have been estimated as described in the Supplementary Materials and set to D_M_ = 100 and D_T_ = 70 μm^2^/s, respectively. A global minimization routine yielded the best diffusion coefficients for each experiment (Figure 4A), whose average values are <D_M_> ≈ 110 ± 5 and <D_T_> ≈ 64 ± 2 μm^2^/s, for the monomeric and dimeric species, respectively.

**Figure 5:**
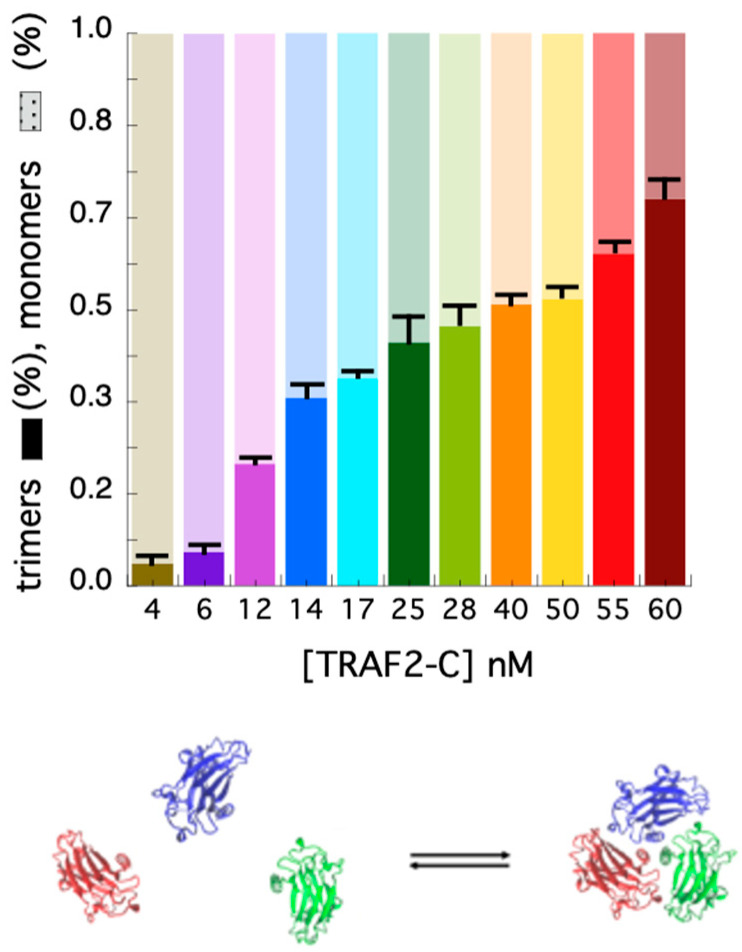
Fraction of monomers (light colors) and trimers (opaque colors) obtained at each TRAF2-C concentration from PCH fits. At the bottom, a schematic representation of the TRAF2-C equilibrium process is shown.

In addition to the autocorrelation analysis, we also used the photon counting histogram approach [45] to determine the fractional contribution of monomers and trimers to the total number of proteins in solution, at different TRAF2-C concentrations. The PCH procedure consists of the analysis of the amplitude of fluorescence fluctuations, which depends on the particles number in the observation volume and on their molecular brightness [45]. In particular, a photon counting histogram is built to report the frequency at which a certain number of photon counts (K) is observed in a fixed time interval, within the excitation volume. Since the association of TRAF2-C protein subunits yields particles characterized by three times the brightness of the monomeric species, the PCH analysis is particularly suitable to discriminate the monomers from oligomers, independently of their diffusion coefficients [45]. The results of the PCHs approach for TRAF2-C in the concentration range 4–60 nM are reported in Figure 4D (diffusion coefficients), Figure 4E (PCHs), and Figure 4F (residuals of two component fitting function, see later). The most relevant feature of this analysis is the broadening of the distribution profile induced at higher particle concentrations (Figure 4E). Such effect is due to an increased frequency of the highest number of counts (i.e., in the range 5 < K < 8), diagnostic of progressive molecular aggregation [45]. Such trend can be used to evaluate changes in the particle distribution (monomers–trimers), interpolating the PCHs values with a two-components fitting function. The result of this analysis is reported in Figure 5. At very low concentration (4–6 nM), the fraction of trimers lays between 5 and 15%, confirming the high dissociation propensity that TRAF2-C displays in aqueous solution [26] due to the dynamics of its subunits [34]. Indeed, a ten times higher concentration (≥40 nM) must be reached to achieve a preponderance of trimers (i.e., ≥50%), as suggested by the last two columns of Figure 5.

### 3.3. Analysis of Secondary, Tertiary, and Quaternary Structure Changes upon Subunit Dissociation

**Figure 6:**
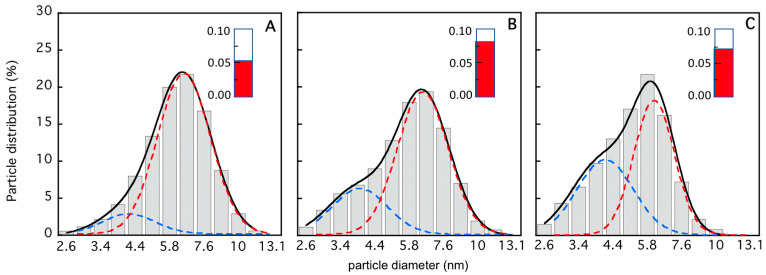
Light scattering particle distribution profile as a function of the particle for [TRAF2-C] ≈ 4 μM (**A**), [TRAF2-C] ≈ 0.4 μM (**B**), and [TRAF2-C] ≈ 0.1 μM (**C**). The data in each histogram have been fitted according to a double gaussian distribution (black, solid line) whose components are reported as dashed lines (blue, for monomers, and red, for trimers). In the insets (red columns), the sample dispersity values are reported (cfr. Section 2).

The characterization of the TRAF2-C dissociation process has been extended from the nanomolar to the micromolar concentration range using dynamic light scattering, circular dichroism, and SANS measurements. The light scattering distribution profiles at three different protein concentrations are reported in Figure 6. The data indicate that the particle distribution becomes more asymmetric upon dilution and less monodisperse. A deconvolution analysis performed using a bimodal gaussian function suggests that at 4 μM the protein is mostly in its trimeric state, the monomers’ contribution being less than 10% (Figure 6A). A larger heterogeneity is instead present in the more diluted samples (Figure 6B,C), with an increase in fraction of the molecular species with a lower molecular weight (up to ≈40%, Figure 6C).

**Figure 7:**
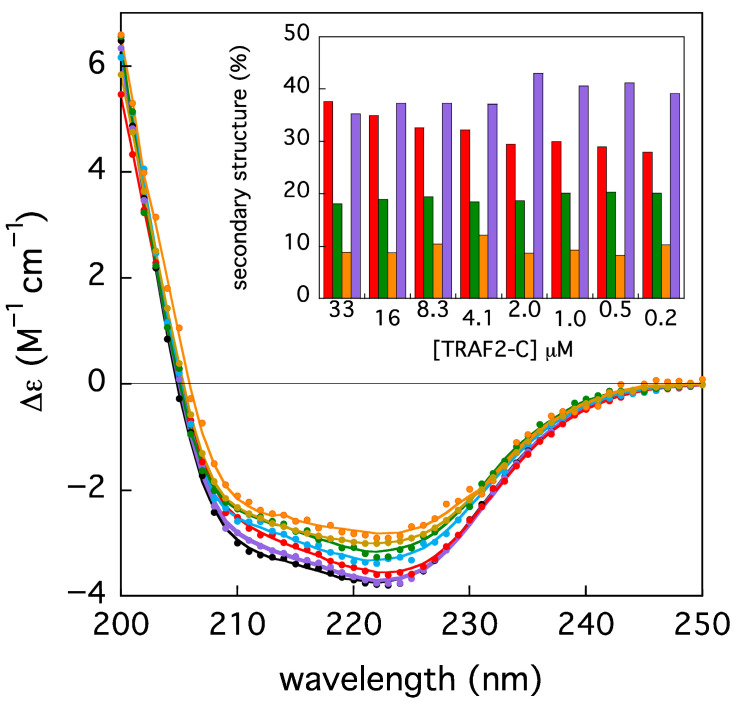
Circular dichroism spectra of TRAF2-C at different concentrations (33, 16, 8, 4, 2, 0.5, and 0.25 μM, from black to orange). The solid lines correspond to the best fits obtained using BeStSel server (cfr. Section 2), whose results are reported as secondary structure content in the histogram in the inset (helix = red, beta = green, turn = orange, and the remains in purple). All spectra and fits have been multiplied by the corresponding dilution factors, for the sake of clarity, with the exception of the initial sample (33 μM, black symbols).

Since the TRAF2-C monomers have been found to be less stable than the trimers both in silico simulations and in vitro measurements [26], the protein folding state has been checked in a series of parallel experiments, monitoring the secondary structure contents upon dilution, through circular dichroism measurements. The ellipticity of TRAF2-C at different concentrations, in the peptide region (200–250 nm), has been reported in Figure 7. The spectra have been re-normalized, considering each dilution factor, with respect to initial, more concentrated sample (i.e., [TRAF2-C] = 33 μM). A partial loss of secondary structure upon subunits dissociation might be envisaged, as re-normalized spectra do not overlap. The intensity changes in the two minima at 310 and 222 nm is not the same (≈−29 and −23%, respectively), indicating that a change in the spectrum shape also occurs (Figure 6). A quantitative analysis of the secondary structure components has been therefore performed with the BeStSel server routine [27]. The results suggest that the most relevant effect is a decrease (≈−20%) in the protein alpha helices content, while the percentages of the other components (beta sheets and turns) oscillate around the initial values (≈18% and 8%, respectively, Figure 7, inset).

**Figure 8:**
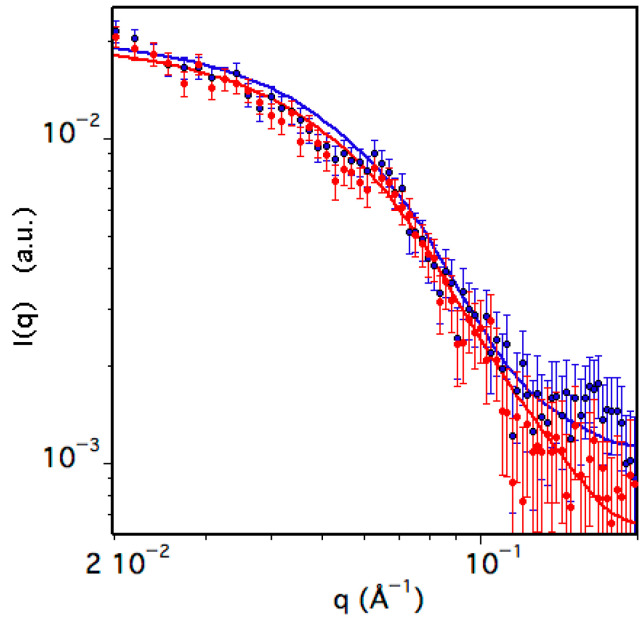
Small Angle Neutron Scattering (SANS) data of TRAF2-C at 20 (blue) and 37 °C (red). The solid lines correspond to the best fit obtained as described in the text and whose parameters are reported in Table 1.

To obtain information on the shape of the species present in solution, Small Angle Neutron Scattering (SANS) measurements of TRAF2-C have been performed. The data have been collected at a protein concentration of about ≈6 μM at two temperatures, namely 20 and 37 °C, and the scattering intensity, I(q), is reported in Figure 8 as a function of the scattering vector. Due to the dissociation equilibrium process, a two-component fitting function was used to fit the data, thus considering both monomeric and trimeric TRAF2-C.

The two species have been approximated with an ellipsoid characterized by two identical radii lying on the equatorial plane and an independent polar radius perpendicular to the others, aligned to the principal axis of rotation. The results, reported in Table 2, indicate that in the monomer the value of the longer radius is 20–25% larger than that of the shorter one (Table 1), thus resembling the shape of a rugby ball. The structure of the trimeric molecule is instead that of a flat, large disk, whose thickness (R_polar_ ≈ 20 Å) is similar to the equatorial section of each monomeric subunit (R_equatorial_ ≈ 18 Å). The percentage of monomers is less than 20% at 20 °C, but it significantly increases to ≈30% at 37 °C (Table 1), indicating that temperature drastically influences the protein quaternary structure.

**Table 1:** Best fit parameters of SANS data.

Molecular Species	R_polar_ (Å)	R_equatorial_ (Å)	N (%)
Mon (20 °C)	24 ± 1	17.6 ± 0.9	18
Trim (20 °C)	20 ± 1	41.3 ± 0.8	82
Mon (37 °C)	23.5 ± 0.6	19 ± 1	28
Trim (37 °C)	18 ± 1	44 ± 2	72

**Table 2:** Best fit of the MD models at ≈ 500 ns using an ellipsoid of revolution (R_1_ × R_2_ × R_3_).

Molecular Species	R_1_ (Å)	R_2_ (Å)	R_3_ (Å)
Monomer	26.9	17.4	20.7
	R_polar_ (Å) ≈ 26.9	^(^*^)^R_equatorial_ (Å) ≈ 19 ± 2	
Trimer	24.7	36.1	40.1
	R_polar_ (Å) ≈ 24.7	^(^*^)^R_equatorial_ (Å) ≈ 38 ± 2	

^(^*^)^ R_equatorial_ was obtained as the mathematical average of the two most similar radii (i.e., R_2_ and R_3_).

### 3.4. A Concerted Evaluation of TRAF2-C Subunits Dissociation Constant

A preliminary investigation into the oligomeric state of TRAF2-C was conducted in vitro a decade ago [26]. This research used an FFS approach to provide evidence that at low concentrations (i.e., ≤50 nM) a strong dissociation of TRAF2-C into subunits occurs. In the present study, we collected a much larger data set, which allowed a more accurate determination of the particle distribution at very low concentration (Figure 5). Above the nanomolar range, molecular fluctuations become difficult to detect using simple photomultipliers, due to a low signal-to-noise ratio [46]. For such reason, we combined light scattering, neutron scattering, circular dichroism, and fluorescence (steady-state and dynamic) data with the FFS results, extending the analysis up to tens of micromolar TRAF2-C (expressed in terms of monomeric subunits).

**Figure 9:**
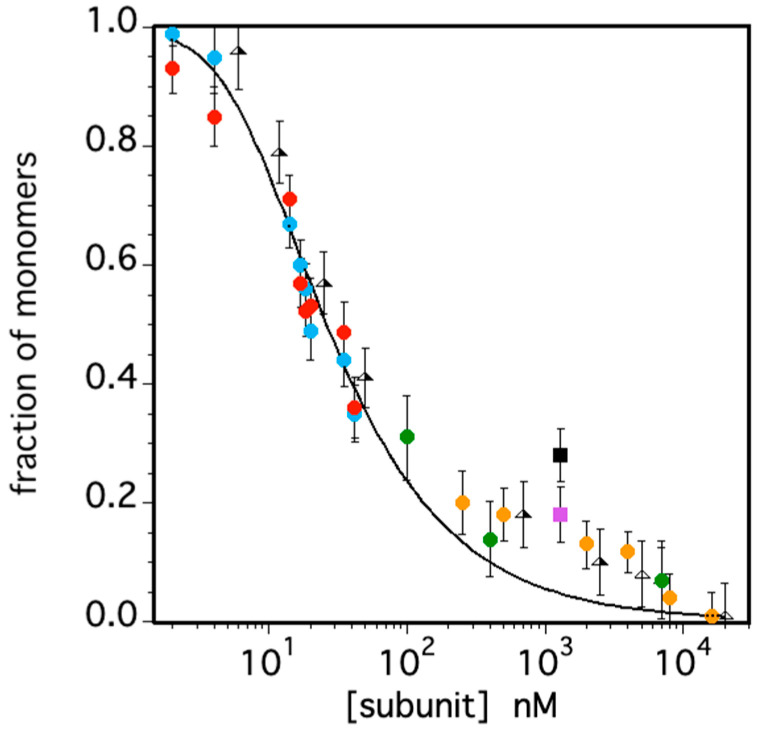
Fraction of monomeric TRAF2-C as a function of subunit concentration as estimated by FFS (cyan and red circles), light scattering (green circles), circular dichroism (yellow circles) neutron scattering (at 20 and 35 °C, pink and black squares, respectively). Previous published data are also reported as triangles. The solid line corresponds to the best fit obtained assuming a simple T ⇆3 M equilibrium between the oligomeric and monomeric protein states as described in [26].

The overall final data set characterizes TRAF2-C dissociation for more than three orders of magnitude, as shown in Figure 8. The results indicate that above 3–4 mM, the percentage of monomers becomes negligeable, while at a few nM, the protein is fully dissociated. Assuming a simple equilibrium between the trimeric (T) and the monomeric (M) species, T ⇆3 M [26], the data fit yielded a dissociation constant of $K_{d}=\left(5.2\;\pm 0.4\right)10^{-16}M^{2}$. Such value is larger than what was previously estimated (≈$1.4\;10^{-16}M^{2}$), using a narrower set of data points [26]. Moreover, the fit yielded an excellent interpolation of the data only below 100 nM or above 5 mM, while between these values the number of monomers seems to be underestimated (Figure 9). A possible explanation of such effect might be due to the presence of dimeric species that, instead, have not been considered both in CD and in light- and neutron-scattering measurements (Figure 6, Figure 7 and Figure 8). In these cases, the introduction of a third molecular species would have required too many parameters, leading to overfitting. On the other hand, pressure-induced dissociation experiments at different protein concentrations have suggested that up to 5% of the total subunits might aggregate to form dimers, at [TRAF2-C] ≈ 2–3 mM [26]. Although this component would give a minor contribution to the overall particle size distribution, the possibility that TRAF2-C might form dimers is reasonable, considering that this protein is known to form functional heterotrimers by combining two TRAF2 subunits with one TRAF1 monomer [3,4]. Indeed, fits of TRAF2-C dissociation using a more complex scheme (i.e., $T_{3}↔D_{2}+M↔3M$) are reported in Supplementary Materials. The results at three different protein concentrations yielded an estimated percentage of dimers ranging from 3 to 11%, at atmospheric pressure.

### 3.5. Molecular Dynamics Prediction of Protein Hydrodynamic Shape: A Contact Network Analysis

**Figure 10:**
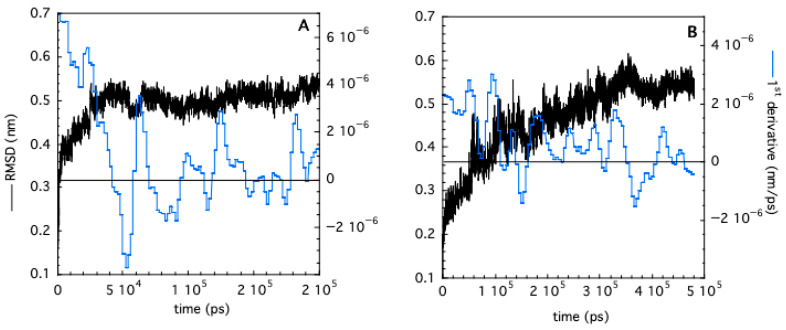
RMSD (black curves) as a function of simulation time obtained in the case of monomeric (**A**) and trimeric (**B**) TRAF2-C. The corresponding first derivatives (evaluated on smoothed RMSD values) are shown in blue.

Molecular dynamics simulations (MD) have been carried out for both monomeric and trimeric TRAF2-C at 20 °C. The time dependence of the RMSD and of its first derivative indicate that the monomer undergoes a very fast conformational transition, reaching equilibrium within 40–50 ns (Figure 10A). This result is in line with previous calculations on the protein subunits’ gyration radius [26]. The dynamics of trimeric TRAF2-C are much slower, and a plateau is reached only after 350 ns (Figure 10B). The different equilibration time interval of the monomer and the trimer suggest that the subunit–subunit interactions are crucial for the overall protein shape and dynamics. Indeed, previous analysis of TRAF2-C dynamic simulations have demonstrated that a “dynamic asymmetry” arises from the reciprocal movement of the three monomers and that the motion of two subunits per time is tightly correlated by key amino acids lying at the monomer–monomer interface [47].

**Figure 11:**
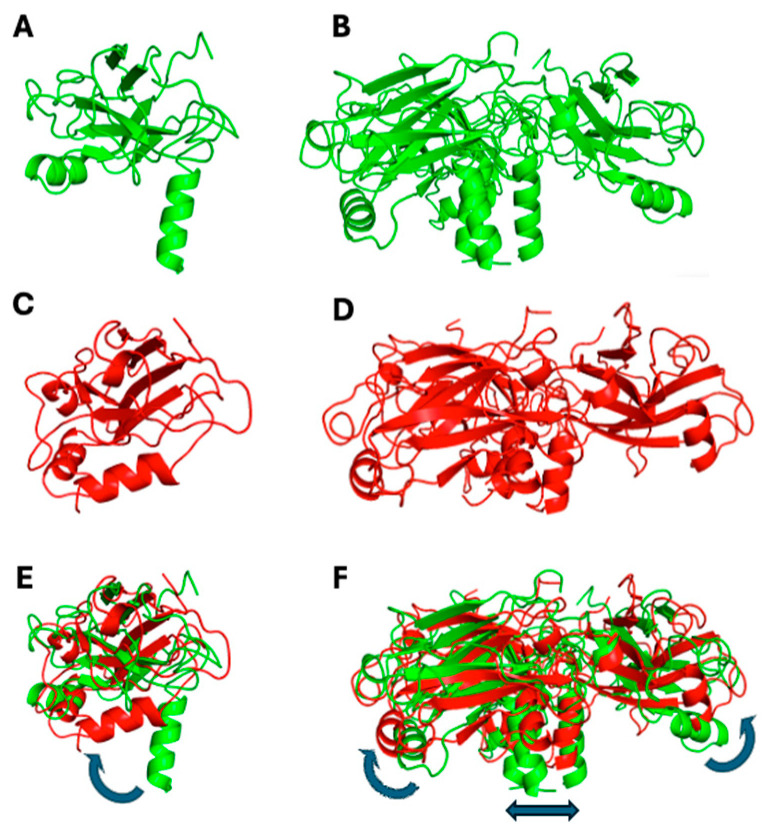
Monomeric (**A**,**C**,**E**) and trimeric (**B**,**D**,**F**) configuration of TRAF2-C in the initial (green) and final (red) frames of the MD simulation (200 and 500 ns for the monomeric and the trimeric protein, respectively). Pictures (**E**,**F**) represent the superposition of each couple of files (initial/final), the blue arrows indicating the major structural re-arrangements.

To compare which structural changes occur during the simulation, cartoons of the initial and final frames are shown in Figure 11, for both monomeric and trimeric species. In the first case, the most striking feature is the bending of the N-terminal alpha helix segment toward the globular protein domain (Figure 11C,E). Such movement takes place (and runs out) in the first 25–30 ns of the simulation, thus accounting for the steep initial change in the monomer’s RMDS (Figure 10A). A partial re-arrangement of the three helices forming the coiled-coil section occurs also in the trimeric TRAF2-C simulation, which is also characterized by the lateral displacement of secondary structure elements belonging to the C-terminal domains (Figure 11D,F).

**Figure 12:**
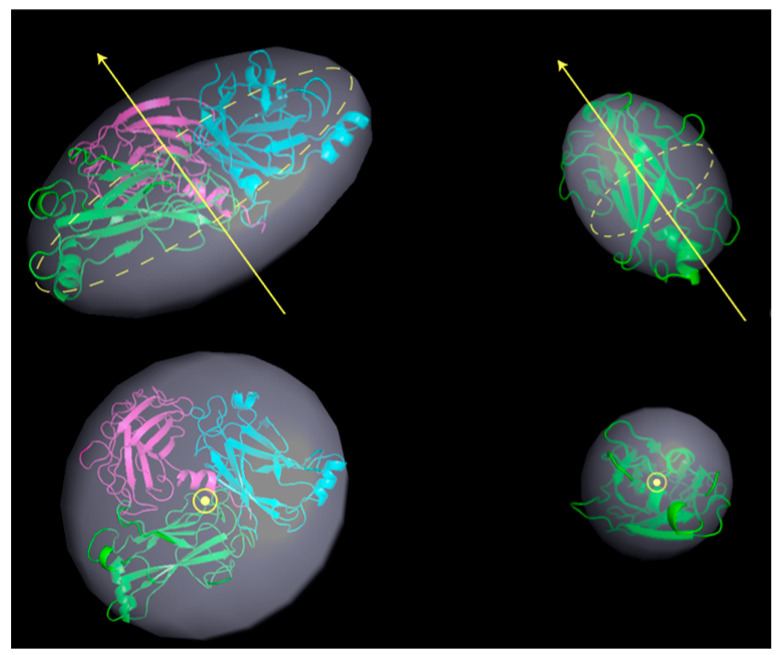
Ellipsoids of revolution (in light gray) for trimeric (left) and monomeric (right) TRAF2-C obtained fitting the MD structures at 500 ns with an ellipsoid of radii R_1_, R_2_, R_3_ (the best fitting values are reported in Table 2). The yellow arrows indicate the principal axis of rotation, aligned with the protein polar radius. The equatorial plane is shown as a yellow, dashed circle. A top view of the ellipsoid is also reported (bottom figures). The cartoon representations of the trimer (left) and monomer (right) at the end of the simulation have been superimposed in each figure.

These changes probably confer a better hydrodynamic shape to the protein tertiary structure in solution, with respect to the crystallographic model. Thus, as a next step, we tried to compare such MD prediction to the experimental results. To this aim, the structures obtained at ≈500 ns (TRAF2-C trimer) or 200 ns (TRAF2-C monomer) have been fitted with the equation of a classical ellipsoid of revolution, and the corresponding three radii are reported in Table 2. Sketches of the protein trimeric and monomeric spheroids are shown in Figure 11 (light gray, transparent shadows) and superimposed to the model obtained with MD. The experimental (Table 1) and theoretical (Table 2) values of the polar and equatorial radii differ from 5 to 10% in the case of the monomer and from 10 to 15% in the case of the trimer. Both the MD model and the SANS results suggest that the trimer correspond to an oblate ellipsoid, whose principal axis of rotation is perpendicular to the larger, equatorial diameters (Figure 12, left panels). The monomer resembles, instead, a prolate object (for instance a rugby ball), whose principal rotation occurs around a longitudinally directed axis (Figure 12, right panels).

Since the equilibrium between the monomer and the protein oligomeric state is strictly dependent on the interaction at the TRAF2-C subunit interfaces, the structures obtained in the simulation have also been characterized through a protein–protein contact network analysis. This approach essentially consists of the search of interconnections among the amino acids of one or more polypeptide chains [39]. Depending on the number of contacts and on their spatial distribution, it is possible to group protein residues into clusters, that is distinct topological units, whose features may evolve and change in time. A spectral clustering rendering is reported in Figure 12 at three steps of MD simulation.

**Figure 13:**
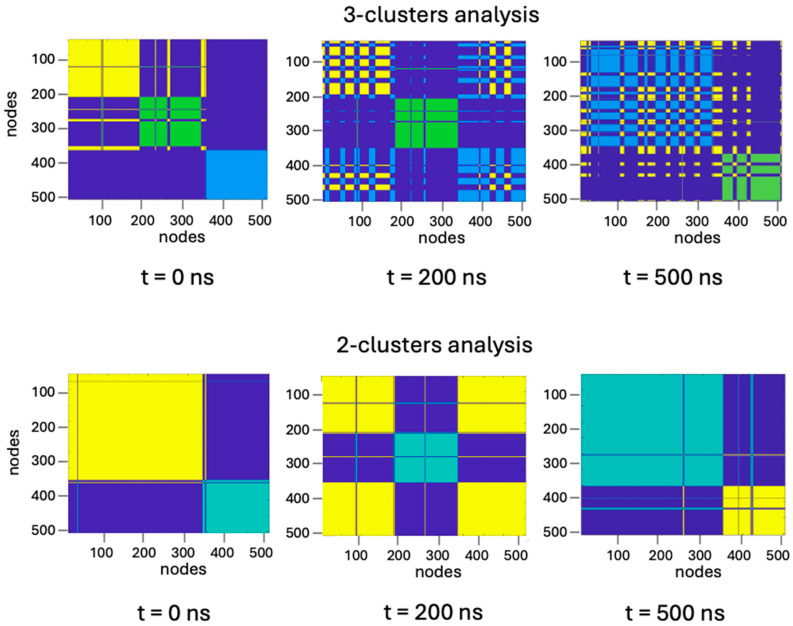
Three-cluster (upper panels) and two-cluster (lower panels) representation of trimeric TRAF2-C at initial (t = 0 ns), intermediate (t = 200 ns), and final (t = 500 ns) steps of simulation time. Each color represents a protein cluster which, in the three-cluster representation, roughly corresponds to a protein subunit. The overlap of two different colors (i.e., where fringes are formed) represents a strong interconnection between two clusters that, in such case, are working as a one.

As the oligomeric state includes three monomers, we started our analysis considering three separate clusters (Figure 13, upper panels), supposing that each cluster is coincident with each subunit. Since the very beginning of the simulation, two monomers (A and B) resulted to be more inter-connected to each other, the third one being “isolated” from the other two, as suggested by the fringes formed in the upper panel (t = 0) shown in Figure 13. In contrast, at t = 200 ns, the two more inter-connected partners become subunits A and C, while at the end of the simulation, the cluster formed by A and B shows again the largest number of intersections (Figure 13 upper panels). Such behavior has been observed under different conditions (for instance changing temperature) in all MD studies on TRAF2-C performed so far [34,47], suggesting that an asymmetrical interaction takes place. The 2:1 dynamic association of the three monomers suggests that a three-cluster analysis is probably redundant. Indeed, the results of a two-cluster analysis (Figure 13, lower panels) yielded similar results (two subunits acting as a single unit), demonstrating that system overstatement can be avoided grouping the protein residues in only two classes.

**Figure 14:**
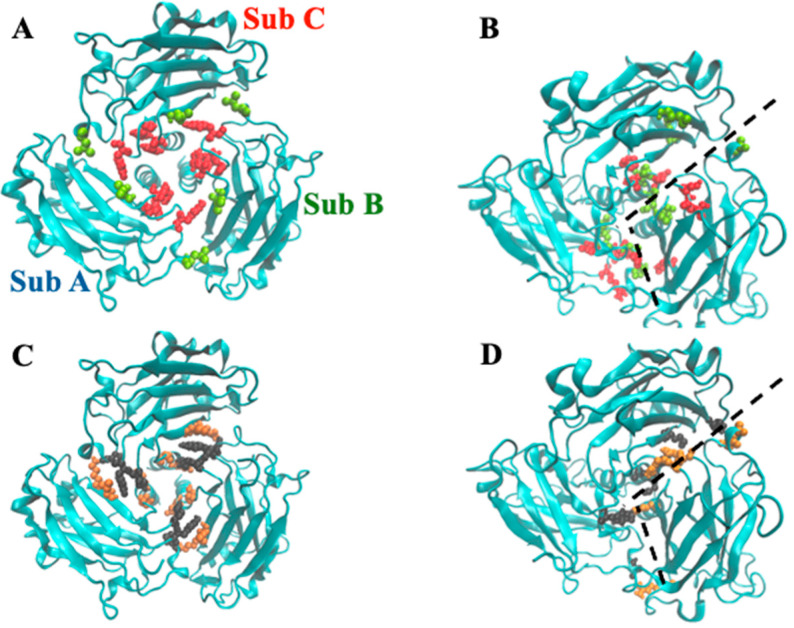
Graphical rendering of TRAF2-C residues (**A**) characterized by a high BC (red, BC > 5000) or a medium BC (lime, 4000 < BC < 5000) in the trimeric crystal structure. In (**B**), the same features are reported at t = 200 ns. In (**C**,**D**), the residues involved in inter-chain crystallographic H-bonds (**C**) or inter-cluster H-bonds ((**D**), t = 200 ns) are reported (black vs. orange), as evaluated by the PISA software v1.48 [49]. The dashed lines in panel B and D correspond to the boundary between cluster AC and subunit B.

To obtain further and more quantitative insights on the inter-chains communication, we have also evaluated the so-called betweenness centrality (BC), a descriptor that features the number of shortest paths crossing a graph node [39]. In the case of proteins, such nodes are represented by the amino acids and, therefore, it is possible to obtain a graphical representation of the BC values associated with each protein conformation as a function of time, using the frames obtained with MD. The application of such procedure to the crystallographic file yielded the result reported in Figure 14 (panel A). In this case, the residues with high BC values (in lime and red) are concentrated in the trimer core and are symmetrically arranged. Recently, it has been shown that TRAF2-C quaternary interactions strongly depend on the pH of the medium [25]. In particular, switching from pH 7.6 to 5.5 induces subunit dissociation [25], suggesting that electrostatic interaction at the monomers’ interface might play a crucial role also in TRAF2-C clustering. In the crystal, the position of the amino acids responsible for inter-chain hydrogen bonds occupy roughly the same positions of the residues characterized by large BC values, yet they do not completely overlap (Figure 14A,C). During the simulation, the 2:1 clustering process obviously changes the arrangement of the residues along the monomers’ interface. Nonetheless, the distribution points characterized by the highest connectivity and the location of the quaternary H-bonds are similar during the protein dynamics, as shown, for instance, in the frame obtained at t = 200 ns (Figure 14B,D). Such feature would suggest that the residues responsible for inter-chain H-bonds play a critical role both in the monomer-trimer equilibrium, upon a significant pH change, and in the TRAF2-C dynamic clustering, upon subunits’ reciprocal movements. In oligomeric structures, the residues with high betweenness values are known to be fundamental for the communication pathways between subunits [48]. The case of trimers, such as TRAF2-C, is rather peculiar because they can assume a “closed ring” configuration in which each subunit interacts with two different partners through distinct interfaces. Local structural changes on one interface are expected to stretch immediately the other two, thus producing changes on the protein surface. Some TRAF2 activities, such as TRADD and TNRF receptors recognition, occur at the level of its C-terminal domain (TRAF2-C), whose shape is therefore critical for the protein function. It is therefore tempting to speculate that, besides the monomer–trimer equilibrium, some of TRAF2 biological features are indirectly modulated by a few central residues, which are closely related to interchain H-bonds.

## 4. Conclusions

The signaling pathway mediated by Tumor Necrosis Factor Receptors (TNFRs) critically depends on trimeric protein structures. Most TNFR ligands are oligomers, composed of three identical subunits. Receptor activation occurs when three TNFR molecules oligomerize within the lipid bilayer, a process that is predominantly driven by ligand binding, but that can also occur through a ligand-independent association of TNFR subunits [50]. On the cytoplasmic side, TNFR-associated factors (TRAFs), such as TRAF2, propagate the signal further, also functioning as trimeric assemblies. TRAF2’s trimerization involves both its C-terminal globular domain—which directly or indirectly interacts with the receptor—and its coiled-coil tail. Thermodynamic studies have shown that TRAF2 exhibits low affinity for monomeric receptor units, supporting the hypothesis that significant conformational rearrangements occur upon binding to trimeric TNFRs [51]. Understanding the oligomerization of the TRAF2-C domain, and the resulting surface changes upon subunit association, is essential for elucidating TRAF2’s mechanism of action—and may provide insights into the broader TRAF family. In the present study, the protein C-terminal oligomerization state has been investigated in detail, using a combination of different experimental approaches that have given insights on the structural features of both monomeric and trimeric species. The parallel investigation of the protein dynamics through in silico simulations and the comparison with the experimental data has opened the possibility to make some hypothesis about the way association/dissociation and clustering is indirectly controlled by residues located in the protein core.

A recent molecular dynamics study on a longer TRAF2 fragment [19] has provided further promising information, suggesting that the TRAF2 tail might also contribute to regulating the conformational changes occurring in the protein globular sections. Extending these studies to the full protein structure is the next frontier of TRAFs scientific research, which is mandatory to face the protein dynamic behavior in vivo.

## Data Availability

The raw data supporting the conclusions of this article will be made available by the authors on request due to the large volume and file size of the data.

## Supplementary Materials

The following supporting information can be downloaded at: https://www.mdpi.com/article/10.3390/biom15111626/s1, Table S1. theoretical hydrodynamic radius and diffusion coefficient of TRAF2-C; Figure S1. Experimental diffusion coefficients reported in literature for proteins of different size (in aqueous buffer using different methodologies) and those found in our lab using FCS; Figure S2. Normalized fluorescence change under pressurization and dimers percentage of TRAF2-C at three different protein concentrations. References cited in Supplementary Materials [26,52,53].

## Abbreviations

The following abbreviations are used in this manuscript:

TRAFs	TNF Receptor-Associated Factors
TRAF-C	TRAF Carboxy-Terminal Domain
TRAF-N	TRAF Amino-Terminal Domain
MD	Molecular Dynamics
FFS	Fluorescence Fluctuations Spectroscopy;
FCS	Fluorescence Correlation Spectroscopy
CD	Circular Dichroism
DLS	Dynamic Light Scattering
SANS	Small Angle Neutron Scattering
FD	Fractal Dimension
PCN	Protein Contact Network
PCH	Photon Counting Histogram
BC	Betweenness Centrality
