# Supplementary material for: Evaluating the Topological Features of Monomeric and Trimeric TRAF2-C: A Multi-Disciplinary Approach
Source: Biomolecules. 2025 Nov 19;15(11):1626. doi: 10.3390/biom15111626 (PMC12650658; doi:10.3390/biom15111626)
Supplement: Supplementary file 1 [file biomolecules-15-01626-s001.zip › biomolecules-3959740-supplementary.pdf]

Supplementary Materials

Theoretical and empirical estimation of TRAF2-C diffusion coefficients

The diffusion coefficient D, of a biological molecule, is strictly related to the protein hydrodynamic radius, R_H, through the Stokes-Einstein equation:

$$D = \frac{K_B T}{6\pi\eta R_H} \tag{S1}$$

where K_B, T and η represent the Boltzmann constant, the temperature, and the viscosity of the medium, respectively. For a perfect spherical particle R_H is obviously linked to the hydrated volume through the relationship:

$$V_H = \frac{4}{3}\pi R_H^3 \tag{S2}$$

The hydrated volume of a protein may be in turn estimated using the equation:

$$V_H = \frac{M}{N_0}\left(\bar{V}_p + \delta\ \bar{V}_w\right) \tag{S3}$$

where N_0 is Avogadro's number, $\bar{V}_p$ is the protein partial specific volume (typically ≈ 0.7 $cm^3\ g^{-1}$), $\bar{V}_w$ is the water specific volume (i.e. 1 $cm^3\ g^{-1}$), while δ is an hydration coefficient (the grams of water molecules bound to a gram of protein) that varies between 0.3 and 0.4 [1]. The substitution of these values in equation (3) (using M_m ≈ 20,000 and M_T ≈ 60,000 for monomeric and trimeric TRAF2-C, respectively) and the combination with equations (1) and (2) yields the expected ranges for the protein hydrated radii and diffusion coefficients reported in Table I. An alternative, possible estimation of the protein radius and, as consequence, of the diffusion coefficient can be obtained using the number of amino acids (Wilkins et al., 1999) from the relationship:

$$R_H = 4.75\ n_{a.a.}^{0.29} \tag{S4}$$

whose values are also reported in Table SI.

To check whether these values are compatible with those found from experiments, we have reported in Figure S1 a plot of the diffusion coefficient found in literature for proteins of different size, in aqueous medium. Most of the experimental data points are below the respective values obtained from the theory, indicating that globular proteins undergo a slower dynamic, because they are not ideal spheroidal objects [2]. On the average, the deviation of the theoretical values from the experimental data is ΔD ≈ -15%, thus more realistic values of monomeric and trimeric TRAF2-C diffusion coefficients should fall within the two ranges reported in the third column of Table SI (as obtained from the data reported in inset of Figure S1).

Table S1. theoretical hydrodynamic radius and diffusion coefficient of TRAF2-C.

	**Using Equations (1)–(3)**		**Using Equation (4)**		**From Figure 4**
	R_H (Å)	D (μm²/s)	R_H (Å)	D (μm²/s)	D (μm²/s)
Monomer 168 a.a.	20–21	116–121	21	115	95–105
Trimer 504 a.a.	28–29	81–83	28.9	83	60–70

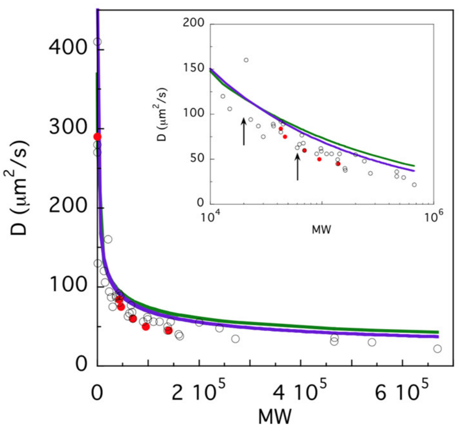

Figure S1. Experimental diffusion coefficients reported in literature for proteins of different size (in aqueous buffer using different methodologies) and those found in our lab using FCS (red). The solid lines correspond to the values calculated using equations 1–3 (purple) or 4 (green) as mentioned in the text. In the inset, the details of same plot are reported using a logarithmic scale for molecular weights (X-axis) in the range 10^4–10^6. The two arrows correspond to the expected position for monomeric and trimeric TRAF2-C.

Estimation of the TRAF2-C dimer fraction in solution

In Figure S2, we report the analysis of the equilibrium dissociation process of TRAF2-C under high pressure, as monitored by fluorescence measurements. The data previously published in [3] were fitted by considering the presence of dimers according to the following scheme:

$$T_3 \leftrightarrow D_2 + M \leftrightarrow 3M$$

With this procedure, the fraction of monomers at 1 atm and at these 3 protein concentrations is lower than those reported in Figure 9 (triangles), where we considered the equilibrium $T_3 \leftrightarrow 3M$ as previously reported [3]

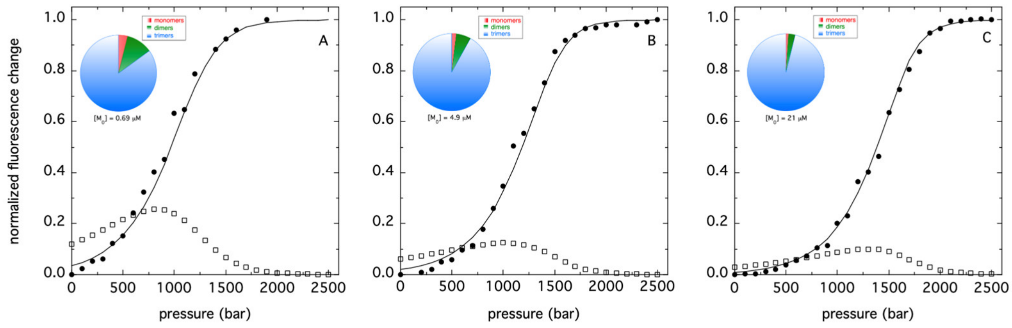

Figure S2. Normalized fluorescence change (filled symbols) under pressurization and dimers percentage (opens symbols) of TRAF2-C at three different protein concentrations. The solid lines represent the best fits obtained using a three steps model as described in the text. In the insets, the initial (i.e. p = 1 bar) percentage of monomers, dimers and trimers are reported.

References

1. Cantor, C.R.; Schimmel, P.R. *Biophysical Chemistry*; W. H. Freeman & Company: New York, NY, USA, 1980.
2. Vasanthi, R.; Bhattacharyya, S.; Bagchi, B. Anisotropic diffusion of spheroids in liquids: Slow orientational relaxation of the oblates. *J. Chem. Phys.* **2001**, *116*, 3.
3. Ceccarelli, A.; Di Venere, A.; Nicolai, E.; De Luca, A.; Minicozzi, V.; Rosato, N.; Caccuri, A.M.; Mei, G. TNFR-Associated Factor-2 (TRAF2): Not Only a Trimer. *Biochemistry* **2015**, *54*, 6153–6161.
